# (*R*)-α-Lipoic acid inhibits fructose-induced myoglobin fructation and the formation of advanced glycation end products (AGEs) in vitro

**DOI:** 10.1186/s12906-017-2076-6

**Published:** 2018-01-15

**Authors:** Hardik Ghelani, Valentina Razmovski-Naumovski, Rajeswara Rao Pragada, Srinivas Nammi

**Affiliations:** 1 School of Science and Health, Western Sydney University, Sydney, NSW 2751 Australia; 2 National Institute of Complementary Medicine (NICM), Western Sydney University, Sydney, NSW 2751 Australia; 3 South Western Sydney Clinical School, School of Medicine, University of New South Wales, Sydney, NSW 2052 Australia; 4 Department of Pharmacology, College of Pharmaceutical Sciences, Andhra University, Visakhapatnam, Andhra Pradesh India

**Keywords:** (*R*)-α-Lipoic acid, Fructose, Fructation, Myoglobin

## Abstract

**Background:**

Fructose-mediated protein glycation (fructation) has been linked to an increase in diabetic and cardiovascular complications due to over consumption of high-fructose containing diets in recent times. The objective of the present study is to evaluate the protective effect of (*R*)-α-lipoic acid (ALA) against fructose-induced myoglobin fructation and the formation of advanced glycation end products (AGEs) in vitro.

**Methods:**

The anti-glycation activity of ALA was determined using the formation of AGEs fluorescence intensity, iron released from the heme moiety of myoglobin and the level of fructosamine. The fructation-induced myoglobin oxidation was examined using the level of protein carbonyl content and thiol group estimation.

**Results:**

The results showed that co-incubation of myoglobin (1 mg/mL), fructose (1 M) and ALA (1, 2 and 4 mM) significantly inhibited the formation of AGEs during the 30 day study period. ALA markedly decreased the levels of fructosamine, which is directly associated with the reduction of AGEs formation. Furthermore, ALA significantly reduced free iron release from myoglobin which is attributed to the protection of myoglobin from fructose-induced glycation. The results also demonstrated a significant protective effect of ALA on myoglobin oxidative damages, as seen from decreased protein carbonyl content and increased protein thiols.

**Conclusion:**

These findings provide new insights into the anti-glycation properties of ALA and emphasize that ALA supplementation is beneficial in the prevention of AGEs-mediated diabetic and cardiovascular complications.

## Background

Protein glycation is a chemical reaction process between a free amino group of a protein and a carbonyl group of a reducing sugar to form a freely reversible Schiff’s base that further rearranges into a more stable intermediary structure called Amadori product [1]. The Amadori product then undergoes oxidative cleavage, generating dicarbonyl compounds to form cross-linked structures termed advanced glycation end products (AGEs). The gradual formation of AGEs in various body tissues play a vital role in further cross-linking or modifying other intracellular and extracellular proteins and in generating reactive oxygen species (ROS) [2]. Hence, the excessive formation of AGEs and their accumulation in various tissues is responsible for age-related diseases [3] and the development of long-term diabetic complications [4].

Myoglobin, a heme-protein expressed in striated muscles (cardiac myocytes and skeletal muscle fibres) plays an important role in the storage and transport of molecular oxygen for cellular respiration [5–9]. Furthermore, myoglobin acts as an intracellular scavenger of nitric oxide (NO), regulating its level in the cardiac muscle and thereby protecting mitochondrial respiration [10–12]. In a high glucose and/or fructose environment, the amino group of myoglobin readily undergoes a non-enzymatic reaction which results in its structural and functional modification in vitro [13, 14]. Furthermore, prolonged incubation of myoglobin with glucose or fructose produces fructosamine followed by AGEs formation [15, 16]. Glucose and fructose-mediated glycation also induces oxidative modification of myoglobin by generating protein carbonyl compounds which may be associated with oxidative stress [13, 17]. During vigorous repeated physical exercise or muscle injury- related disorder such as rhabdomyolysis produce muscle damage releasing myoglobin into circulation, where myoglobin comes in contact with circulatory glucose and/or fructose [18]. Free myoglobin in circulation can undergo glycation reactions specifically in the poor glycaemic situation and may be a source of various pathophysiological complications such as diabetic ketoacidosis and renal failure [19, 20]. However, intracellular myoglobin glycation in muscles cell, particularly in hyperglycaemic condition and its implications in the development of complications, is still not known.

Although many synthetic and natural anti-glycation compounds have been evaluated in vivo and in vitro, no single compound effectively suppresses protein glycation in a clinical setting. Aminoguanidine has been shown to be a potent inhibitor of the protein glycation process and fluorescent AGEs formation in animals and in humans [21]. However, its clinical use is limited due to severe adverse effects such as flu-like symptoms, gastrointestinal problems and anaemia [22–24]. Therefore, there is an urgent need to evaluate new compounds that inhibit protein glycation and thus may be beneficial in preventing diseases mediated by AGEs.

(*R*)-α-Lipoic acid (ALA; 1,2-dithiolane-3-pentanoic acid), also known as thioctic acid, is traditionally recognized as an essential cofactor in mitochondrial respiratory enzymes that catalyse the oxidative decarboxylation reactions [25]. Chemically, ALA is a short-chain fatty acid with a disulfide group in its dithiolane ring and a chiral carbon resulting in *R* and *S* enantiomers. Although the majority of the commercially produced ALA consists of a racemic admixture, the *R* form is the biologically active form that is endogenously produced by the body, while the *S* form is produced from chemical manufacture and is not biologically active [26]. At the cellular level, ALA is reduced to dihydrolipoic acid (DHLA), which has a number of cellular actions including free radical scavenging and modulating oxidative stress and inflammatory pathways [26]. ALA, when exogenously administered, is readily absorbed from the gut and has been clinically used in Europe for the treatment of diabetic polyneuropathy [27].

ALA has been shown to possess various pharmacological properties including anti-oxidant, anti-inflammatory, detoxifying, antidiabetic, cardiovascular, anti-ageing, anticancer, cognitive and neuroprotective [28, 29]. In laboratory experiments, the effect of ALA on protein glycation and AGEs formation has been investigated both in vitro and in vivo. Dietary supplementation of ALA in rats fed chronically with glucose significantly decreased mitochondrial superoxide in the heart and AGEs formation in the aorta [30]. Furthermore, chronic supplementation of ALA in fructose-fed-rats significantly attenuated AGEs-mediated skin-collagen cross-linking and other physicochemical abnormalities [31]. In addition, chronic treatment of ALA in high-fructose fed rats markedly lowered glucose, glycated protein, glycated hemoglobin and fructosamine in circulation; enhanced in vitro glucose utilization with prevention of glycation and accumulation of AGEs in isolated rat diaphragm [32]. In obese Zucker rats, chronic treatment of ALA significantly inhibited protein carbonyls content and improved insulin sensitivity in skeletal muscle [33]. ALA also inhibited AGEs production and down-regulated the receptor for advanced glycation end products (RAGE) expression in streptozotocin-induced diabetic rats [34] and in human embryonic kidney cells and in rat sensory neurons [35, 36]. In addition, topical treatment of ALA nanoparticles significantly down-regulated the expression of RAGE and enhanced cutaneous wound healing in streptozotocin-induced diabetic mice [37].

In an in vitro glycation model containing bovine serum albumin and glucose, ALA markedly inhibited fructosamine, protein carbonyls and fluorescent AGEs production [38, 39]. Moreover, ALA markedly suppressed AGEs-induced activation of NF-kB in cultured vascular endothelial cells [40] and in retinal endothelial cells [41]. In another independent study, exogenous administration of ALA diminished AGEs-induced endothelial expression of vascular cell adhesion molecule-1 (VCAM-1) and monocyte binding to endothelium [42]. Furthermore, ALA prevented the up-regulation of AGEs-induced inducible nitric oxide synthase (iNOS) expression and nitric oxide (NO) production in murine microglial cells [43]. ALA also reduced the AGEs-mediated formation of lipid peroxidation products in human neuronal cells [44, 45] and in rat cortical neurones [46]. However, ALA has not been examined in myoglobin glycation induced by fructose. Hence, in the present study, we investigated the effects of ALA on fructose-induced myoglobin fructation and AGEs formation.

## Methods

### Chemicals and reagents

(*R*)-α-Lipoic acid, myoglobin, nitro blue tetrazolium (NBT), hydroxylamine hydrochloride, ferrozine, dinitrophenylhydrazine (DNPH), guanidine hydrochloride, ethyl acetate, ethanol, trichloroacetic acid (TCA), dimethyl sulfoxide (DMSO), fructose, 5,5′-dithio-bis (2-nitrobenzoic acid) (DTNB), L-cysteine and aminoguanidine (AG) were purchased from Sigma-Aldrich (St. Louis, MO, USA). Fructosamine and iron standards were obtained from PM Separations (Capalaba DC, QLD, Australia). All other chemicals and reagents used were of analytical grade.

### Evaluation of myoglobin fructation inhibitory effect of α-lipoic acid under fructose overload in vitro

Myoglobin fructation was performed according to the methods previously described by Roy and colleagues with minor modification [13, 16]. Briefly, 500 μL of myoglobin (final concentration: 1 mg/mL) was incubated with 400 μL of fructose (final concentration: 1 M) solution at 37 °C in the dark for up to 30 days in the presence or absence of ALA (100 μL; dissolved in DMSO) at a final concentration of 1, 2 and 4 mM. Aminoguanidine (100 μL; dissolved in DMSO), at a final concentration of 5 mM was used as a positive control. After the specified incubation period (10, 20 or 30 days), aliquots of the glycated reaction mixtures were assayed for fluorescent AGEs, free iron, fructosamine (glycated protein), protein carbonyls and protein thiols.

#### Determination of fluorescent AGEs formation

The formation of fluorescent AGEs in the reaction mixture after 30 days of incubation was measured according to the method of Wrobel et al. [46]. Briefly, to 1 mL of the reaction mixture, 250 μL of TCA (100%) was added. The resulting mixture was vortexed for 60 s and centrifuged in a refrigerated centrifuge (Biofuge Stratos, Thermo Scientific) at 14,000 rpm for 4 min. The supernatant was collected in a disposable polystyrene cuvette, and fluorescence intensity was read at an excitation wavelength 355 nm and emission wavelength 460 nm using a spectrofluorometer (Wallac 1420 Victor 3 V, Perkin Elmer). The percentage inhibition of fluorescent AGEs formation was calculated as follows:

$$ \mathrm{Inhibition}\  \mathrm{of}\  \mathrm{fluorescent}\  \mathrm{AGEs}\ \left(\%\right)=\left[\left(\mathrm{Fluorescence}\  \mathrm{intensity}\  \mathrm{of}\  \mathrm{control}-\mathrm{Fluorescence}\  \mathrm{intensity}\  \mathrm{of}\  \mathrm{sample}\right)/\mathrm{Fluorescence}\  \mathrm{intensity}\  \mathrm{of}\  \mathrm{control}\right]\times 100 $$

#### Estimation of free iron in fructated myoglobin (ferrozine test)

The free iron, as a measure of fructation-induced iron release in the reaction mixture after 10, 20 and 30 days of incubation, was estimated according to the method of Panter [47]. Briefly, to 250 μL of the reaction mixture, 250 μL of ice cold TCA (20%) was added and centrifuged in a refrigerated centrifuge at 15,000 rpm for 4 min. To 250 μL of the supernatant, 2.5 mL of iron buffer (1.5% hydroxylamine hydrochloride in acetate buffer, pH 4.5) and 50 μL iron colour reagent (0.85% ferrozine in iron buffer) were added. The resultant mixture was incubated at 37 °C for 30 min and the absorbance was measured at 560 nm using a UV-visible spectrophotometer (Ultrospec 2100 Pro*,* Biochrom). The concentration of liberated free iron was calculated as follows:

$$ {\displaystyle \begin{array}{l}\mathrm{Concentration}\  \mathrm{of}\  \mathrm{free}\  \mathrm{iron}\ \left(\upmu \mathrm{g}/\mathrm{dL}\right)=\\ {}\left(\mathrm{Absorbance}\  \mathrm{of}\  \mathrm{test}/\mathrm{Absorbance}\  \mathrm{of}\  \mathrm{standard}\right)\mathrm{X}\;\mathrm{Concentration}\  \mathrm{of}\  \mathrm{standard}\;\left(\upmu \mathrm{g}/\mathrm{dL}\right)\end{array}} $$

#### Estimation of fructosamine (fructated myoglobin)

The concentration of fructosamine, as a measure of glycated protein in the reaction mixture after 10, 20 and 30 days of incubation was measured according to the method of Ohkawara et al. [48]. Briefly, to 250 μL of the reaction mixture, 1 mL of 0.5 mM NBT reagent (in carbonate buffer; pH 10.8) was added in a disposable polystyrene cuvette inside a UV-visible spectrophotometer. The absorbance difference at 540 nm after 10 min and 15 min was used to calculate the formation of fructosamine using the following formula:

$$ {\displaystyle \begin{array}{l}\mathrm{Concentration}\  \mathrm{of}\  \mathrm{fructosamine}\ \left(\upmu \mathrm{M}\right)=\\ {}\left(\mathrm{Absorbance}\  \mathrm{of}\  \mathrm{test}\ \mathrm{at}\ 15\mathrm{th}\ \min \hbox{--} \mathrm{Absorbance}\  \mathrm{of}\  \mathrm{test}\ \mathrm{at}\ 10\mathrm{th}\ \min \right)/\\ {}\left(\mathrm{Absorbance}\  \mathrm{of}\  \mathrm{standard}\ \mathrm{at}\ 15\mathrm{th}\ \min \hbox{--} \mathrm{Absorbance}\  \mathrm{of}\  \mathrm{standard}\ \mathrm{at}\ 10\mathrm{th}\ \min \right)\ X\  Concentration of standard\ \left(\mu M\right)\end{array}} $$

#### Estimation of protein carbonyls content

The level of protein carbonyls, as a measurement of fructation-induced protein oxidation in the reaction mixture after 10, 20 and 30 days of incubation, was estimated according to the method of Levine et al. [49]. Briefly, to 200 μL of the reaction mixture, 200 μL of 10 mM DNPH (in 2.5 M hydrochloric acid) was added. After thorough mixing, 250 μL of TCA (30%) was added and centrifuged in a refrigerated centrifuge at 15,000 rpm for 4 min. The pellet was collected and washed three times with 1 mL ethanol: ethyl acetate (1:1) mixture to remove any unreacted DNPH. The pellet was then dissolved in 1 mL of 6 M guanidine hydrochloride (in 20 mM phosphate buffer, pH 6.6), incubated at 37 °C for 15 min and centrifuged in a refrigerated centrifuge at 15,000 rpm for 4 min. The absorbance of the supernatant was then measured at 375 nm using a UV-visible spectrophotometer. The concentration of protein carbonyls was expressed as nanomoles of carbonyls per milligram of protein using the molar absorption coefficient of DNPH (22,000 M^−1^ cm^−1^).

#### Estimation of free protein thiols

The concentration of free protein thiols, as a measure of fructation-induced antioxidant defence in the reaction mixture after 10, 20 and 30 days of incubation, was measured by Ellman’s assay [50] with minor modifications. Briefly, 70 μL of the reaction mixture was incubated with 130 μL of 5 mM DTNB (in 0.1 M phosphate buffered saline) at room temperature for 15 min and the absorbance was measured at 412 nm using a UV-visible spectrophotometer. The concentration of protein thiols was calculated using a standard curve of L-cysteine and expressed as nanomoles of L-cysteine per milligram of protein.

#### Statistical analysis

The results were expressed as a means ± SD (*n* = 6). To examine the quantitative differences among the experimental groups, the respective data were subjected to one-way analysis of variance (ANOVA) using GraphPad Prism-5.0 (GraphPad Software Inc., California, USA) statistical programme. Post hoc comparisons were made using Dunnett’s multiple comparison test. Statistical differences in individual groups at different time points were detected using Student’s paired t-test. In all tests, *p* < 0.05 was used as the criterion for statistical significance.

## Results

### Effect of α-lipoic acid on fluorescent AGEs formation

**Fig. 1:**
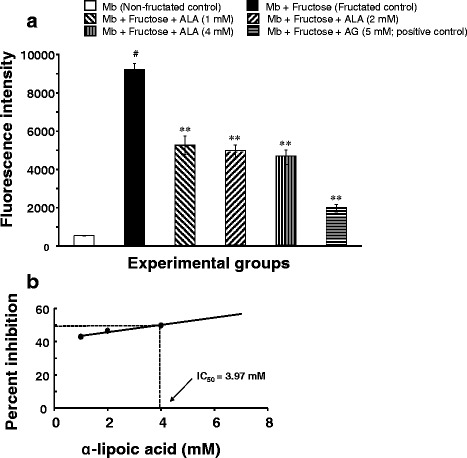
Effect of α-lipoic acid on the formation of fluorescent AGEs in fructose-induced myoglobin fructation. Each bar represents the mean ± SEM in six separate experiments (*n* = 6). Significant difference from fructated control: **p < 0.01. Significant difference from non-fructated control: #*p* < 0.001. ALA = (R)-α-lipoic acid; AG = Aminoguanidine; Mb = Myoglobin

The effect of ALA on the formation of fluorescent AGEs in myoglobin-fructose glycation system was observed on day-30 of incubation. As shown in Fig. 1a, incubation of myoglobin with fructose (fructated control) significantly (*p* < 0.001) increased the formation of fluorescent AGEs by 17-fold as shown by increased fluorescence intensity (9226.7 ± 398.4 vs 539.7 ± 4.8) compared with myoglobin incubation alone (non-fructated control). On the other hand, ALA co-treatment in the myoglobin-fructose glycation system at 1, 2 and 4 mM concentrations elicited significant (*p* < 0.01) concentration-dependent inhibition of fluorescent AGEs formation with a maximum reduction of 49.7% (4641.7 ± 590.2 vs 9226.7 ± 398.4) at 4 mM concentration compared with the fructated control. The concentration of ALA required to inhibit 50% (IC_50_) of fluorescent AGEs as determined from linear regression analysis was found to be 3.97 mM (Fig. 1b). In comparison, co-treatment of aminoguanidine (a known inhibitor of glycation process; positive control) at a concentration of 5 mM in myoglobin-fructose glycation system produced a significant (*p* < 0.01) 78.6% inhibition of fluorescent AGEs formation (1969.8 ± 276.0 vs 9226.7 ± 398.4) compared with the fructated control.

### Effect of α-lipoic acid on free iron release

**Fig. 2:**
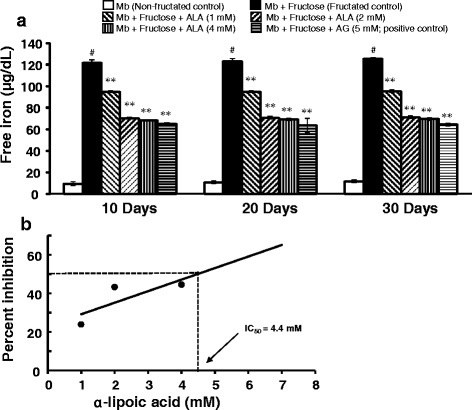
Effects of α-lipoic acid on free iron release in fructose-induced myoglobin fructation. Each bar represents the mean ± SEM in six separate experiments (n = 6). Significant difference compared to fructated control at identical times: **p < 0.01. Significant difference from non-fructated control: ^#^*p* < 0.001. ALA = α-lipoic acid; AG = Aminoguanidine; Mb = Myoglobin

Figure 2a shows the effect of ALA on fructation-induced iron release in myoglobin-fructose glycation system as observed on day-10, 20 and 30 of incubation. A significant (*p* < 0.001) 13-fold increase in free iron (121.8 ± 5.3 vs 9.4 ± 2.2) was observed on day-10 when myoglobin was co-incubated with fructose (fructated control) compared with myoglobin incubation alone (non-fructated control) and moreover, this difference was consistent throughout the study period. Nevertheless, no significant time-dependent change in free iron level was observed in the myoglobin-fructose co-incubation on day-20 or day-30 compared with day-10 value. However, ALA co-treatment in myoglobin-fructose glycation system at 1, 2 and 4 mM concentrations significantly (*p* < 0.01) displayed a concentration-dependent reduction in free iron levels on day-10 compared with the fructated control and moreover, this difference was consistent throughout the study period. On day-30 of co-treatment, ALA significantly (*p* < 0.01) decreased the free iron levels with a maximum reduction of 44.5% (69.5 ± 2.9 vs 125.3 ± 1.1) at 4 mM concentration compared with the fructated control. Nevertheless, at all the studied ALA concentrations, no significant time-dependent change in free iron levels was observed within groups on day-20 or day-30 compared with day-10 values. The concentration of ALA required to inhibit 50% (IC_50_) of free iron release as determined from linear regression analysis was found to be 4.4 mM (Fig. 2b). In comparison, co-treatment of aminoguanidine (a known inhibitor of glycation process; positive control) at a concentration of 5 mM in myoglobin-fructose glycation system produced a significant (*p* < 0.01) reduction in free iron release on day-10 compared with the fructated control which was consistent throughout the study period, achieving a maximum 48.8% reduction (64.2 ± 2.4 vs 125.3 ± 1.1) on day-30.

### Effect of α-lipoic acid on fructosamine formation

**Fig. 3:**
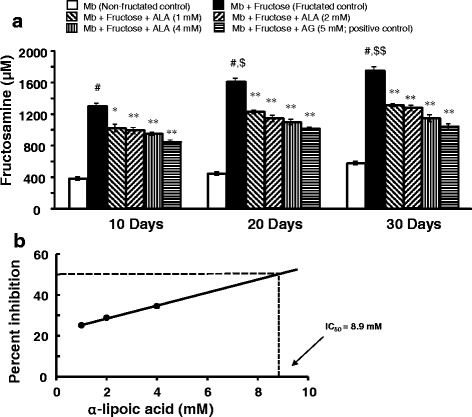
Effects of α-lipoic acid on fructosamine formation in fructose-induced myoglobin fructation. Each bar represents the mean ± SEM in six separate experiments (n = 6). Significant difference compared to fructated control at identical times: * *p* < 0.05; **p < 0.01. Significant difference from non-fructated control: ^#^*p* < 0.001. Significant difference from the respective Day-10 value: ^$^*p* < 0.05; ^$$^*p* < 0.01. ALA = α-lipoic acid; AG = Aminoguanidine; Mb = Myoglobin

The effect of ALA on fructosamine formation in myoglobin-fructose glycation system as observed on day-10, 20 and 30 of incubation is shown in Fig. 3a. A significant 3-fold increase in fructosamine (1300.2 ± 72.5 vs 486.4 ± 39.4; *p* < 0.001) was observed on day-10 when myoglobin was co-incubated with fructose (fructated control) compared with myoglobin incubation alone (non-fructated control) and moreover, this difference was more pronounced after day-20 (1608.8 ± 84.1 vs 557.4 ± 23.3; *p* < 0.001) and day-30 (1754.8 ± 83.2 vs 564.9 ± 51.8; *p* < 0.001) of the study. Moreover, a marked time-dependent increase in fructosamine was observed in myoglobin-fructose co-incubation that gained significance by day-20 (1608.8 ± 84.1 vs 1300.2 ± 72.5; *p* < 0.05) and day-30 (1754.8 ± 83.2 vs 1300.2 ± 72.5; *p* < 0.01) compared with day-10. On the other hand, ALA co-treatment in the myoglobin-fructose glycation system at 1, 2 and 4 mM concentrations significantly (*p* < 0.05 to *p* < 0.01) displayed a concentration-dependent reduction in fructosamine levels on day-10 compared with the fructated control and moreover, this difference was consistent throughout the study period. On day-30 of co-treatment, ALA showed a significant (*p* < 0.01) decrease in fructosamine levels with a maximum reduction of 34.5% (1275.9 ± 66.4 vs 1754.8 ± 83.2) at 4 mM concentration compared with the fructated control. Nevertheless, at all the studied ALA concentrations, no significant time-dependent change in fructosamine levels was observed on day-20 or day-30 compared with day-10 values. The concentration of ALA required to inhibit 50% (IC_50_) of fructosamine as determined from linear regression analysis was found to be 8.9 mM (Fig. 3b). In comparison, co-treatment of aminoguanidine (a known inhibitor of glycation process; positive control) at a concentration of 5 mM in myoglobin-fructose glycation system produced a significant (*p* < 0.01) reduction in fructosamine on day-10 compared with the fructated control, which was consistent throughout the study period, achieving a maximum 40.6% reduction (1088.8 ± 69.7 vs 1754.8 ± 83.2) on day-30.

### Effect of α-lipoic acid on protein carbonyls formation

**Fig. 4:**
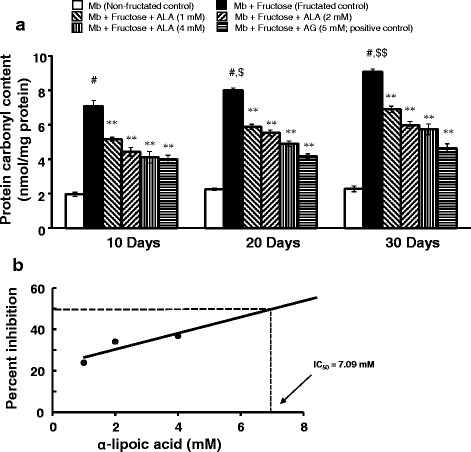
Effect of α-lipoic acid on protein carbonyl content in fructose-induced myoglobin fructation. Each bar represents the mean ± SEM in six separate experiments (*n* = 6). Significant difference compared to fructated control at identical times: * *p* < 0.05; **p < 0.01. Significant difference from non-fructated control: ^#^*p* < 0.001. Significant difference from the respective Day-10 value: ^$^*p* < 0.05, ^$$^*p* < 0.01. ALA = α-lipoic acid; AG = Aminoguanidine; Mb = Myoglobin

Figure 4a depicts the effect of ALA on protein carbonyls formation in myoglobin-fructose glycation system as observed on day-10, 20 and 30 of incubation. A significant 3.6-fold increase in protein carbonyls (7.1 ± 0.4 vs 2.0 ± 0.2; *p* < 0.001) was observed on day-10 when myoglobin was co-incubated with fructose (fructated control) compared with myoglobin incubation alone (non-fructated control) and moreover, this difference was more pronounced after day-20 (8.01 ± 0.2 vs 2.3 ± 0.2; *p* < 0.001) and day-30 (9.1 ± 0.2 vs 2.3 ± 0.2; *p* < 0.001) of the study. Furthermore, a marked time-dependent increase in protein carbonyls was observed in myoglobin-fructose co-incubation that gained significance by day-20 (8.0 ± 0.2 vs 7.1 ± 0.4; *p* < 0.05) and day-30 (9.1 ± 0.2 vs 7.1 ± 0.4; *p* < 0.01) compared with day-10. On the other hand, ALA co-treatment in the myoglobin-fructose glycation system at 1, 2 and 4 mM concentrations significantly (*p* < 0.01) displayed a concentration-dependent reduction in protein carbonyls levels on day-10 compared with the fructated control and moreover, this difference was consistent throughout the study period. On day-30 of co-treatment, ALA showed a significant (*p* < 0.01) decrease in protein carbonyls levels with a maximum reduction of 41.9% (5.7 ± 0.4 vs 9.1 ± 0.2) at 4 mM concentration compared with the fructated control. Nevertheless, at all the studied ALA concentrations, no significant time-dependent change in protein carbonyls levels was observed on day-20 or day-30 compared with day-10 values. The concentration of ALA required to inhibit 50% (IC_50_) of protein carbonyls as determined from linear regression analysis was found to be 7.09 mM (Fig. 4b). In comparison, co-treatment of aminoguanidine (a known inhibitor of glycation process; positive control) at a concentration of 5 mM in myoglobin-fructose glycation system produced a significant (*p* < 0.01) reduction in protein carbonyls on day-10 compared with the fructated control which was consistent throughout the study period achieving a maximum 48.7% reduction (4.7 ± 0.4 vs 9.1 ± 0.2) on day-30.

### Effect of α-lipoic acid on protein thiols oxidation

**Fig. 5:**
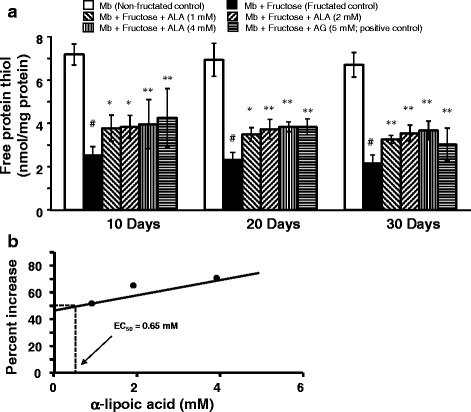
Effect of α-lipoic acid on protein thiol oxidation group in fructose-induced myoglobin fructation. Each bar represents the mean ± SEM in six separate experiments (n = 6). Significant difference compared to fructated control at identical times: * *p* < 0.05; **p < 0.01. Significant difference from non-fructated control: ^#^*p* < 0.001. ALA = α-lipoic acid; AG = Aminoguanidine; Mb = Myoglobin

The effect of ALA on free protein thiols in myoglobin-fructose glycation system as observed on day-10, 20 and 30 of incubation is depicted in Fig. 5a. A significant 2.8-fold decrease in free protein thiols (2.6 ± 0.2 vs 7.2 ± 0.8; *p* < 0.001) was observed on day-10 when myoglobin was co-incubated with fructose (fructated control) compared with myoglobin incubation alone (non-fructated control) and moreover, this difference was consistent throughout the study period. Nevertheless, no significant time-dependent change in free protein thiols was observed in the myoglobin-fructose co-incubation on day-20 or day-30 compared with day-10 value. On the other hand, ALA co-treatment in the myoglobin-fructose glycation system at 1, 2 and 4 mM concentrations significantly (*p* < 0.05 to *p* < 0.01) displayed a concentration-dependent increase in free protein thiols levels on day-10 compared with the fructated control and moreover, this difference was consistent throughout the study period. On day-30 of co-treatment, ALA showed a significant (*p* < 0.01) increase in free protein thiols levels with a maximum increment of 68.1% (3.7 ± 0.4 vs 2.2 ± 0.7) at 4 mM concentration compared with the fructated control. Nevertheless, at all the studied ALA concentrations, no significant time-dependent change in free protein thiols levels was observed on day-20 or day-30 compared with day-10 values. The concentration of ALA required to increase 50% (EC_50_) of free protein thiols as determined from linear regression analysis was found to be 0.65 mM (Fig. 4b). In comparison, co-treatment of aminoguanidine (a known inhibitor of glycation process; positive control) at a concentration of 5 mM in myoglobin-fructose glycation system produced a significant (*p* < 0.01) increase in free protein thiols on day-10 compared with the fructated control which was consistent throughout the study period achieving a maximum 36.3% increase (3.0 ± 1.9 vs 2.2 ± 0.7) on day-30.

## Discussion

Protein glycation, also known as the Maillard reaction, is a complex biochemical reaction beginning with the non-enzymatic interaction of a reducing sugar with an amino group of protein [51]. Molecular rearrangements (Schiff base formation and Amadori rearrangements) lead to the formation of AGEs that trigger pathogenic signalling pathways and cross-link extracellular matrix proteins [52]. In recent decades, over consumption of high-fructose diets has dramatically increased and has been linked to an increase in obesity and diabetic complications [53]. Furthermore, in the hyperglycaemic condition, the polyol pathway becomes active and facilitates the conversion of intracellular glucose to fructose. In the context of intracellular protein glycation, the rate of fructose-induced glycation is faster than glucose-induced glycation and thus, fructose and its metabolites are considered as important precursors in the intracellular formation of AGEs [54–56].

Myoglobin is an oxygen-binding heme-protein localized in oxidative muscle (i.e. cardiac myocardium and skeletal muscle fibres) and functions to transport and store oxygen, scavenge nitric oxide and reactive oxygen species [57]. Recently, it has been demonstrated that myoglobin non-enzymatically reacts with reducing sugars and generates various AGEs [13, 14]. Furthermore, it has also been shown that fructose has a greater rate of myoglobin glycation and AGEs formation than glucose [56, 58, 59]. Recently, Banerjee and Chakraborti [60, 61] demonstrated that myoglobin, when incubated with methylglyoxal, a known AGEs precursor, induced structural modifications of myoglobin and generated various AGEs. Studies have shown that many antioxidant-rich natural products have the ability to prevent reducing sugar-mediated protein glycation [62, 63]. In this study, we examined the preventive effects of ALA in fructose-induced myoglobin glycation (fructation) and AGEs formation using an in vitro glycation model. The clinical pharmacokinetic characteristics of ALA (200 mg/day to 600 mg/day) have been widely reported with varied pharmacokinetic profiles in multiple studies as reviewed by Shay and colleagues [26]. The disparity in the observed oral bioavailability and peak plasma concentration has been attributed to whether *R*-enantiomer, its salt form, or a racemic mixture of ALA has been used in the studies, plus any associated formulation and/or biopharmaceutical factors. Hence, has yet to be established a clear relationship between the oral dosage, bioavailability and peak plasma concentration of ALA. Therefore, the selected concentrations of ALA used in this study were rationalised based on our previous experience and on the published literature [64–66]. To our knowledge, this is the first study which investigates the protective effects of α-lipoic acid in fructose-induced myoglobin glycation.

Based on fluorescence property, we examined the influence of ALA on the formation of total AGEs. Our results demonstrated that ALA efficiently inhibited fructose-induced AGEs formation which supports a previous report on the inhibitory effects of ALA on fluorescent AGEs formation in vivo [34, 35, 67]. The possible mechanism of action of ALA in inhibiting the formation of fluorescent AGEs include: (i) blocking the amino groups of protein, thus preventing its glycation with free sugar, (ii) blocking the carbonyl groups of reducing sugars, (iii) preventing the formation of Amadori products by blocking the Schiff’s base to Amadori products conversion, (iv) blocking the Amadori products and dicarbonyl intermediates which may reduce glycation, as well as AGEs formation, and/or (v) preventing autoxidation of fructose and glycoxidation of Amadori products. The protein glycation reaction generates various fluorescent AGEs such as pentosidine and crossline which are implicated in the development of diabetic cardiovascular complications [1, 68]. It has been demonstrated that serum fluorescent AGEs (such as pentosidine) were significantly higher in diabetic patients and was associated with an increased incidence of cardiovascular disease [69, 70]. Moreover, AGEs can cross-link with extracellular matrix proteins such as collagen, thereby increasing arterial wall and myocardial stiffness. This leads to systolic and diastolic dysfunction of the heart and potentiates heart failure in diabetic patients [71]. Furthermore, binding AGEs to its receptor (RAGE) cause modifications to LDL (i.e. oxidation of LDL) and subsequently, generate foam cells, which are hallmarks of atherosclerosis [72]. Therefore, preventing the formation of AGEs or removing cross-linked AGEs is an efficient way of interrupting the glycation cascade and preventing the potential pathological consequences of AGEs.

In our study, ALA also displayed a significant, time-dependent inhibition of fructosamine formation. A possible mechanism for inhibiting the formation of Amadori products could be either by competing with sugar molecules or protecting the protein amino group from the nucleophilic addition of the carbonyl group of the sugar. In the early stages of protein glycation, the reaction between the carbonyl group of sugar and the amino group of protein form freely reversible Schiff’s bases which further rearrange to form more stable ketoamine or Amadori products such as fructosamine [73]. At this stage, the reducing sugar itself undergoes autoxidation in the presence of transition metals and generates various highly reactive superoxide radical and hydroxyl radical. The harmful radicals further accelerate the glycation process to form AGEs. In addition, the Amadori products also react with free protein and generate AGEs [72]. Thus, the reduction of fructosamine would be beneficial in the suppression of AGEs formation and therapeutically delay the occurrence of AGEs-mediated complications.

Furthermore, in this study, myoglobin-fructose glycation effectively released free iron from the heme moiety of myoglobin in a time-dependent manner which is effectively suppressed by ALA. During myoglobin glycation, iron is liberated from the heme and most likely ligated to distal histidine in the heme pocket of myoglobin. This iron termed as “mobile reactive iron” can catalyse the Haber-Weiss reaction producing free radicals (particularly hydroxyl (OH) radicals), which increase cellular oxidative stress and damage different cellular constituents [15, 16, 74]. Roy and co-workers demonstrated that the in vitro, non-enzymatic glycation of myoglobin induces the release of free iron from the heme pocket of myoglobin, and the iron release was found to be proportional to the extent of myoglobin glycation [13].

We also examined the influence of ALA against fructose-mediated non-enzymatic glycation and oxidation-dependent damage to myoglobin. ALA suppressed the formation of protein carbonyls content and oxidation of thiols in this study. ALA is a potent biological antioxidant and is capable of scavenging many free radicals such as hydroxyl groups [75, 76]. During protein glycation, reactive di-carbonyl intermediates and protein carbonyl derivatives generate AGEs formation and also modify protein structure which is prone to oxidative reaction with amino acids such as cysteine, particularly the thiol side chain. The reactive oxygen species and reactive nitrogen species are also generated during glycation and glycoxidation. In the meantime, they also can oxidize side chains of amino acid residues of the protein to form a carbonyl derivative and diminish the oxidative defense of protein by eliminating the thiol groups [77, 78]. These alterations are reflective of oxidative protein damage, with oxidative stress and the formation of AGEs. Therefore, a possible mechanism of ALA in suppressing the formation of protein dicarbonyls is through scavenging the highly reactive free radicals generated during chronic glycation.

## Conclusion

In conclusion, these findings demonstrate that ALA protects against fructose-mediated myoglobin glycation in vitro by inhibiting the early and intermediate glycation reactions involved in the formation of AGEs. Thus, the present findings emphasize that ALA supplementation is beneficial in the prevention of AGEs-mediated diabetic and cardiovascular complications. Further studies are warranted to investigate the ability of ALA on late-stage glycation events that lead to AGEs production and on AGEs-mediated protein cross-linking and cellular signaling pathways.

## Abbreviations

AG: Aminoguanidine
AGEs: Advanced glycation end products
ALA: (*R*)-α-Lipoic acid
DNHP: Dinitrophenylhydrazine
LDL: Low-density lipoproteins
Mb: Myoglobin
NBT: Nitro-blue tetrazolium
RAGE: Receptor for advanced glycation end products
ROS: Reactive oxygen species
TCA: Trichloro acetic acid
